# Pilot study of ziv-aflibercept in myopic choroidal neovascularisation patients

**DOI:** 10.1186/s12886-020-01679-4

**Published:** 2020-10-19

**Authors:** Amin E. Nawar, Heba M. Shafik

**Affiliations:** Department of Ophthalmology, Tanta University, Tanta, Egypt

**Keywords:** Central macular thickness, Choroidal neovascularization, Optical coherence tomography, Pathological myopia, Ziv-aflibercept

## Abstract

**Background:**

Myopic choroidal neovascularization (CNV) is the most common sight-threatening complication associated with high myopia. The present study evaluated the efficacy and safety of the intravitreal injection of ziv-aflibercept in patients with myopic CNV.

**Methods:**

This prospective interventional study was conducted on 20 eyes of 20 patients with active myopic CNV. Twelve patients were 40 years or older. This study was performed in the Ophthalmology Department of Tanta University Eye Hospital, Tanta University, Egypt. Optical coherence tomography (OCT) was performed for all patients at baseline and monthly after injection during the 6-month follow up period. The main outcome measures were changes in BCVA and CMT. The exploratory outcome measures were CNV size, IOP and the number of injections needed in each age group during the study period.

**Results:**

Patients with myopic CNV younger than 40 years needed fewer injections (2.00 ± 0.76) than patients older than 40 years (2.50 ± 1.00), with no statistical significance detected between the two groups (*p*-value 0.246). CNV was smaller in the younger age group (p-value 0.209), best corrected visual acuity (BCVA) improved significantly in the younger and older age groups (*p*-values 0.001 and 0.028, respectively), and central macular thickness (CMT) decreased significantly after 6 months, from 242.88 ± 23.83 μm to 191.13 ± 13.83 μm in the younger age group and from 251.33 ± 26.60 μm to 197.08 ± 17.64 μm in the older age group (*p* = 0.001). No significant correlation was found between the final BCVA and either the spherical equivalent or central macular thickness after 6 months, with *p*-values of 0.135 and 0.145, respectively. No significant changes in IOP were detected in either group after the intravitreal injection.

**Conclusion:**

Ziv-aflibercept is a highly effective and safe drug in cases of active myopic CNV; however, a larger number of patients and a longer follow-up period are needed to confirm our results.

This study was retrospectively registered at clinicaltrials.gov (ID: NCT04290195) on 26-2-2020.

## Background

The prevalence of pathological myopia in adults is approximately 1–3%, and myopic choroidal neovascularisation (CNV) is one of the most sight-threatening complications, occurring in approximately 5–11% of pathological myopia patients [1, 2]. Several treatment approaches have been proposed due to the poor understanding of its pathological nature [2]. Photodynamic therapy (PDT) is one of the treatment options for myopic CNV that can reduce the risk of visual loss, as supported by several studies [3, 4]. However, patients treated with PDT showed limited improvement in the mean visual acuity that did not remain longer than 2 years [5]. Recently, anti-vascular endothelial growth factor (anti-VEGF) agents such as bevacizumab, ranibizumab, and aflibercept were introduced for the treatment of many retinal diseases, with high safety profiles. The off-label use of bevacizumab and the introduction of ranibizumab and aflibercept as treatment modalities for myopic CNV showed promising results in preventing visual deterioration for a long time with minimal side effects [6, 7]. According to the phase 3 RADIANCE trial [8], ranibizumab has been approved for the treatment of visual impairment due to myopic CNV; in addition, MYRROR, a phase 3 study, confirmed the efficacy and safety of intravitreal aflibercept in the treatment of CNV in pathological myopia patients [9].

Ziv-aflibercept (Zaltrap; Regeneron, New York, USA) is a recombinant fusion protein that is very similar to aflibercept. It was approved by the FDA in August 2012 for the treatment of resistant metastatic colorectal carcinoma. Recently, it was reported that off-label intravitreal ziv-aflibercept is an effective and safe treatment at 4-week intervals, without any ocular toxicity in patients with age-related macular degeneration [10].

The present study evaluated the efficacy and safety of ziv-aflibercept as a primary treatment in patients with myopic CNV younger than and older than 40 years.

## Methods

### Study design

This prospective interventional case study was conducted on 20 eyes of 20 patients with active myopic CNV who attended the outpatient clinic of Tanta University Eye Hospital between March 2019 and September 2019 after approval from the Ethical Committee of the Faculty of Medicine, Tanta University, Egypt (approval code 32970/02/19). Patients were divided into two age groups to detect the influence of age on visual prognosis: group 1 included 12 patients ≥40 years old, and group 2 included the remaining eight patients, who were <  40 years old. Sample size calculation was based on the mean (SD) of BCVA pre- and post-treatment with ziv-aflibercept (0.67 (0.45) and 0.58 (0.43), respectively, based on previous research) [11]. G*power version 3.0.10 was used to calculate the difference between 2 paired readings using a 2-tailed t test with an α error = 0.05, a power = 80.0% and an effect size =0.65. The total calculated sample size was 20 cases.

All procedures were carried out under the tenets of the Helsinki Declaration. Written consent was provided by all participants after discussing the procedure, alternative treatment plans, follow-up schedules, and possible benefits and risks. This study was retrospectively registered at clinicaltrials.gov (ID: NCT04290195) on 26-2-2020.

### Participants

This study included treatment-naive patients who were recently diagnosed with active myopic subfoveal or juxtafoveal CNV for less than 2 months that was confirmed by fundus fluorescein angiography (FFA) and optical coherence tomography (OCT). Fifteen cases of subfoveal CNV and five cases of juxtafoveal CNV were included. Pathological myopia patients with a spherical equivalent ≥ − 6 diopters and an axial length of ≥26 mm were enrolled in the current study.

Patients with a history of intraocular surgery, coincident retinal pathology such as diabetic retinopathy, retinal vein occlusion (RVO), CNV due to other causes such as age-related macular degeneration, angioid streaks, trauma, choroiditis and extrafoveal myopic CNV were excluded from the study. In addition, patients who received other lines of treatment for CNV, such as photodynamic therapy, laser photocoagulation or intravitreal injection of triamcinolone or other anti-VEGF agents, and those known to be glaucomatous or have an IOP ≥20 mmHg were also excluded.

Furthermore, patients with other retinal pathologies, such as prior ocular inflammation, retinal degeneration, and dense media opacity, including nuclear sclerosis, and those who did not complete 6 months of follow-up were not enrolled in the present study.

A thorough ophthalmic evaluation, including a BCVA test using a Snellen chart that was converted to LogMAR for statistical analysis, IOP measurements using applanation tonometry, an anterior segment examination using a slit lamp, a posterior segment examination by slit lamp bimicroscopy using a + 78 D lens, and indirect ophthalmoscopy, was performed for all patients. Spectral-domain OCT (Topcon 3D Optical Coherence Tomography) was performed for all patients at baseline and at the postoperative first-month visit and then monthly for 6 months.

### Surgical procedure

#### Preoperative preparation

Patients were prepared by applying topical fluoroquinolone eye drops (moxifloxacin hydrochloride 0.5% Vigamox, Alcon, USA) 4 times daily for 3 days before injection.

#### Procedure

The intravitreal injection was carried out in the operating room under complete aseptic techniques with an operating microscope. After the topical application of anaesthetic drops (benoxinate hydrochloride 0.4%, Benox, Epico, Egypt) to the ocular surface followed by the topical application of 10% povidone-iodine (Betadine) to the periocular area, lids and eye lashes, 5% povidone iodine was administered inside the conjunctival sac for 3 min before the intravitreal injection. Next, 0.05 ml of 1.25 mg of ziv-aflibercept (Zaltrap) was injected into the vitreous cavity in the inferotemporal quadrant of the globe using a 30-gauge needle 4 mm from the limbus.

#### Postoperative care

After injection, topical antibiotic drops (moxifloxacin hydrochloride 0.5% Vigamox, Alcon, USA) were applied, and the eye was patched for several hours. Patients were instructed to administer antibiotic drops four times daily for 3 days. Patients were examined the following day and the third day after injection to exclude any complications, such as an elevated IOP, endophthalmitis, retinal breaks, retinal detachment and vitreous haemorrhage. All patients were followed up at 4-week intervals after the first injection. At each visit, a thorough ophthalmic examination and SD-OCT were performed. The pro re nata (PRN) regimen was followed in this study, in which an additional intravitreal injection of Zaltrap was given after 1 month and monthly for sixth months if there was no improvement in or stabilization of best corrected visual acuity (improvement by more than one line in the Snellen chart), haemorrhage was detected during the clinical examination, and/or persistent intraretinal or subretinal fluid was detected on SD-OCT.

Statistical analysis was conducted using Student’s t-test, the chi-square test, the linear correlation coefficient, and analysis of variance (ANOVA) with the Statistical Package for Social Science (SPSS, Chicago, IL, USA). An unpaired Student’s t-test was used to compare quantitative data between two groups. Data are presented as the mean and standard deviation. The chi-square value indicates that the row and column variables are independent but does not indicate the strength or direction of the relationship.

The Pearson correlation coefficient was used to detect correlations between two quantitative variables in one group. Student’s t test and a paired t test were used to compare 2 continuous parametric variables (2 independent groups and 2 dependent groups, respectively). A *P*-value ≤0.05 was considered significant.

## Results

This study was performed from March 2019 until September 2019. Twenty eyes with active myopic CNV were collected beginning in March 2019, and the baseline demographic data of all patients, including age, sex, spherical equivalent, number of injections and CNV size, were recorded (Table 1).

**Table 1:** Demographic data, spherical equivalent, number of injections and the size of CNV

	Total number of examined eyes ***n*** = 20	Age of the studied cases		Test of significance
		< 40 years *n* = 8	≥ 40 years *n* = 12	
**Age (years)**				
**Range**	**31–63**	**31–39**	**40–63**	***P*** = 0.001*
**Gender n(%)**				
**Male**	**8 (40%)**	**3 (37.5%)**	**5 (41.7%)**	χ^**2**^ **= 0.035**
**Female**	**12 (60%)**	**5 (62.5%)**	**7 (58.3%)**	***P*** **= 0.852**
**Spherical equivalent**				
**Mean ± S. D**	**−7.05 ± 0.52**	**−6.78 ± 0.36**	**−7.23 ± 0.55**	**t = 2.03**
**Range**	**(−8)-(−6.25)**	**(−7.25)–(− 6.25)**	**(−8)–(− 6.25)**	***P*** **= 0.058**
No of injections				
Mean ± S. D	2.30 ± 0.92	2.00 ± 0.76	2.50 ± 1.00	t = 1.198
Range	(1–4)	(1–3)	(1–4)	*p* = 0.246
Size of CNV by disc diameter				
Mean ± S. D	0.65 ± 0.42	0.59 ± 0.37	0.88 ± 0.55	t = 1.30 *p* = 0.209

*No* Number, *S.D* Standard deviation, χ^*2*^ Chi-Square test, *t* Student t test

*p value

### Primary and secondary outcomes

Compared with baseline, BCVA improved significantly after 6 months of injection in both age groups, with a more notable improvement in the younger age group than the older age group, from a mean of 0.88 (0.28) before injection to 0.48 (0.23) (*p* = 0.001) in patients < 40 years old and from a mean of 0.60 (0.21) before injection to 0.33 (0.24) after 6 months (*p* = 0.028) in patients ≥40 years old. The greatest improvement in BCVA was within the first 2 months. These results are shown in Table 2.

**Table 2:** BCVA and CMT at the base line and after 6 months of follow up

	Total number of examined eyes ***n*** = 20	Age of the studied cases		Test of significance (between age groups)
		< 40 years ***n*** = 8	≥ 40 years ***n*** = 12	
**BCVA Before injection**				
Mean ± S. D	0.77 ± 0.28	0.88 ± 0.28	0.60 ± 0.21	t1 = 2.56
Range	0.4–1.4	0.4–1.4	0.4–1	*p* = 0.0197
**BCVA After 6 months**				
Mean ± S. D	0.42 ± 0.24	0.48 ± 0.23	0.33 ± 0.24	t1 = 1.392
Range	0.1–1	0.22–1	0.1–0.7	*p* = 0.181
t2	17.609	14.443	5.998	
***P*** **value**	0.001*	0.001*	0.028*	
**CMT (um) Before injection**				
Mean ± S. D	247.95 ± 25.24	242.88 ± 23.83	251.33 ± 26.60	t1 = 0.724
Range	212–289	212–289	213–287	*p* = 0.478
**CMT (um) After 6 months**				
Mean ± S. D	194.70 ± 16.11	191.13 ± 13.83	197.08 ± 17.64	t1 = 0.802
Range	165–235	165–210	175–235	*p* = 0.433
t2	63.252	28.223	34.663	
***P*** **value**	0.001*	0.001*	0.001*	

*BCVA* Best corrected visual acuity, *CMT* Central macular thickness, *No* Number, *S.D* Standard deviation, *t1* Student t test, *t2* Paired t test

*p value

Also, patients with myopic CNV who were younger than 40 years needed significantly fewer injections (2.00 ± 0.76) than patients who were 40 years or older (2.50 ± 1.00) (*p*-value 0.246). CNV was smaller in the younger age group, with a p-value of 0.209. These results are shown in Table 1.

Furthermore, central macular thickness (CMT) decreased significantly after 6 months, from 242.88 ± 23.83 μm to 191.13 ± 13.83 μm in the younger age group and from 251.33 ± 26.60 μm to 197.08 ± 17.64 μm in the older age group at baseline (*p* = 0.001), as shown in Table 2.

No significant change in IOP after the intravitreal injection was detected in either group after 6 months (*p* = 0.140), as illustrated in Table 3.

**Table 3:** Illustrates changes in IOP after injection

		Before injection	After 6 months	Paired t test
**IOP**	**Mean** ± **SD**	12.53 ± 1.77	13.47 ± 1.60	t = 1.517 *p* = 0.140
	**Range**	10–16	10–16	

*IOP* Intra-ocular pressure

**Fig. 1:**
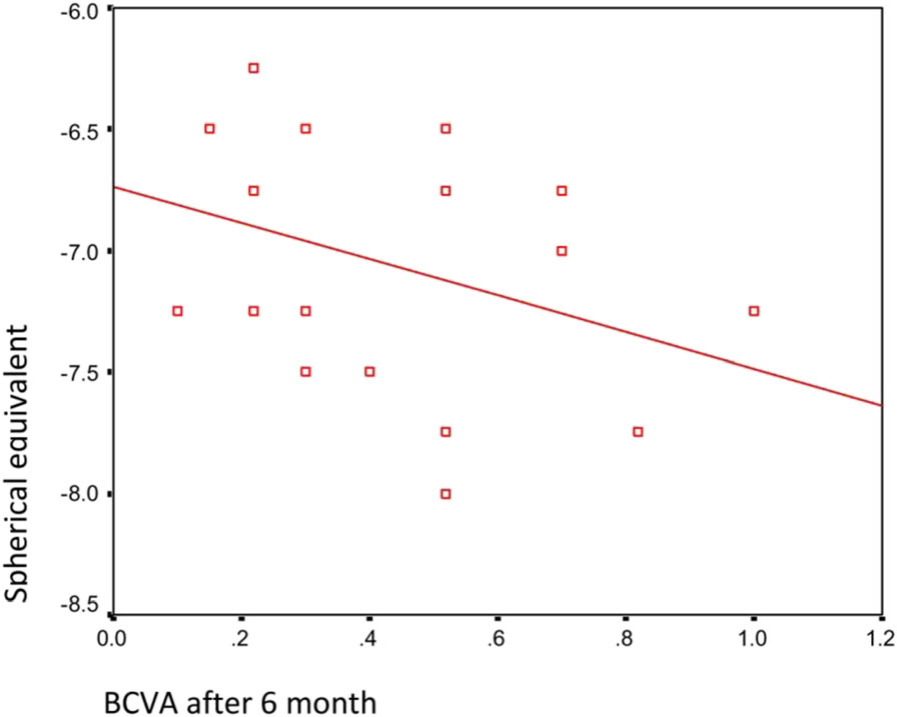
Correlation between the BCVA after 6 months and spherical equivalent in all eyes

**Fig. 2:**
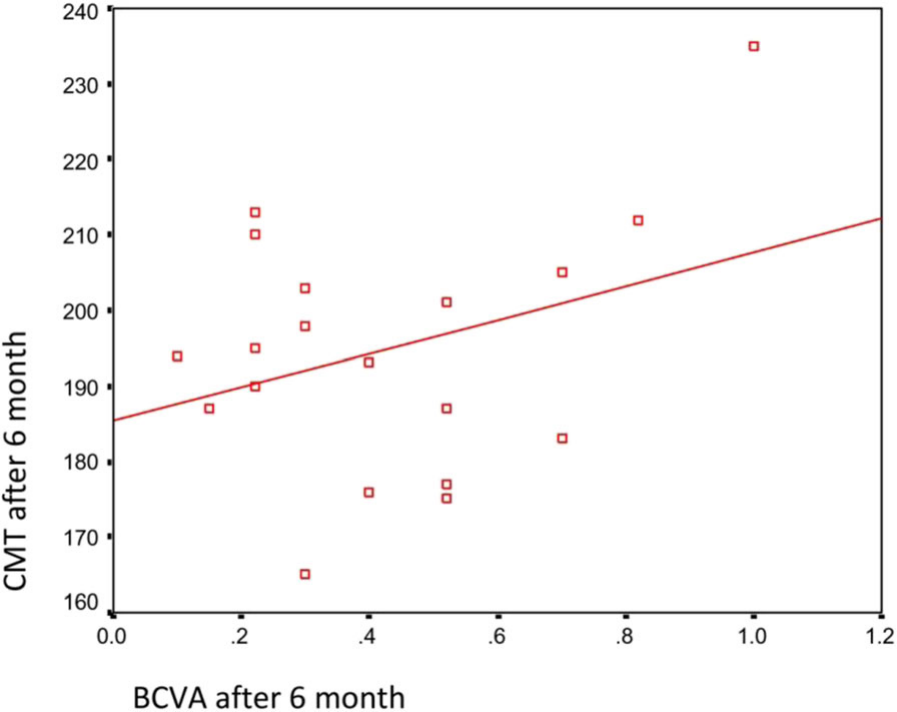
Correlation between the final BCVA and the final CMT after 6 months in all eyes

Additionally, there was no significant correlation between the final BCVA and either the spherical equivalent or the final CMT after 6 months (*p*-values 0.135 and 0.145, respectively) (Table 4, Figs. 1 and 2).

**Table 4:** Correlation between BCVA by logMAR and both the spherical equivalent and CMT after 6 months

	BCVA by log MAR after 6 months	
	r.	*P*
**Total eyes**		
**Spherical equivalent**	- 0.346	0.135
**CMT after 6 months**	0.338	0.145
**< 40 years**		
**Spherical equivalent**	- 0.051	0.905
**CMT after 6 months**	0.027	0.950
**≥ 40 years**		
**Spherical equivalent**	- 0.335	0.440
**CMT after 6 months**	0.286	0.152

*r* Pearson correlation co-efficient, *CMT* Central macular thickness, *BCVA* Best corrected visual acuity

**Fig. 3:**
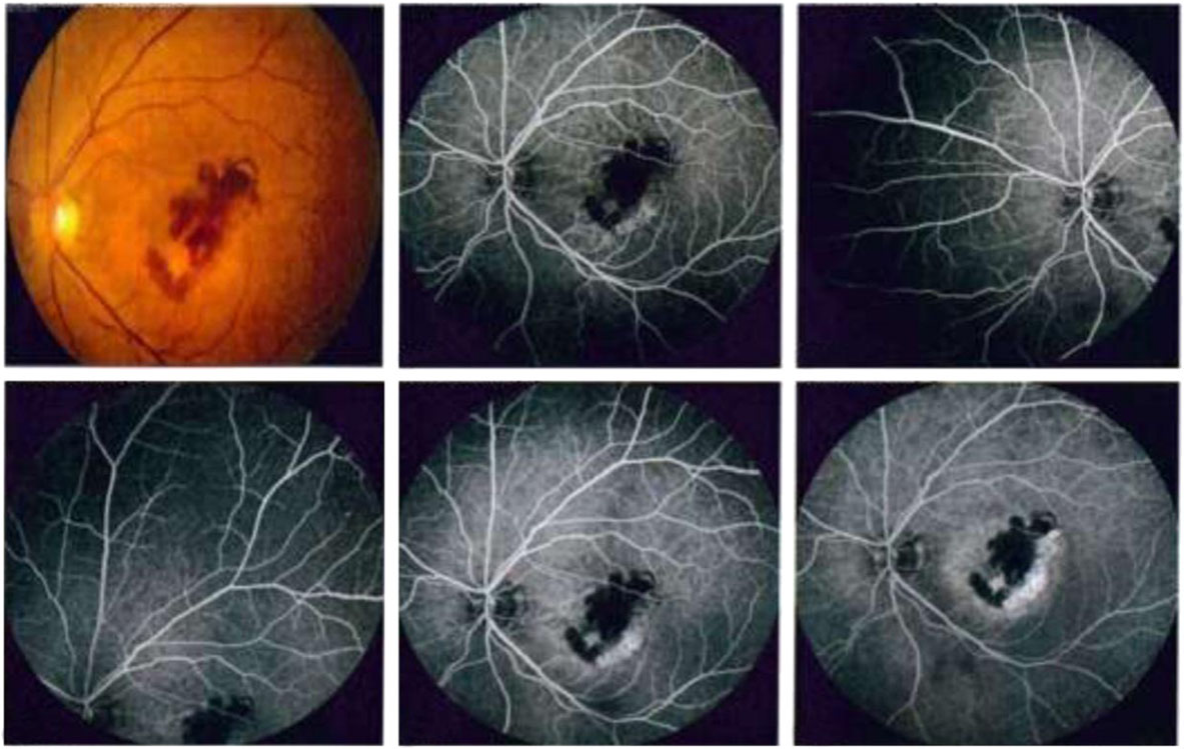
Fluorescein angiography of a female patient 55 years old with left active myopic subfoveal CNV

**Fig. 4:**
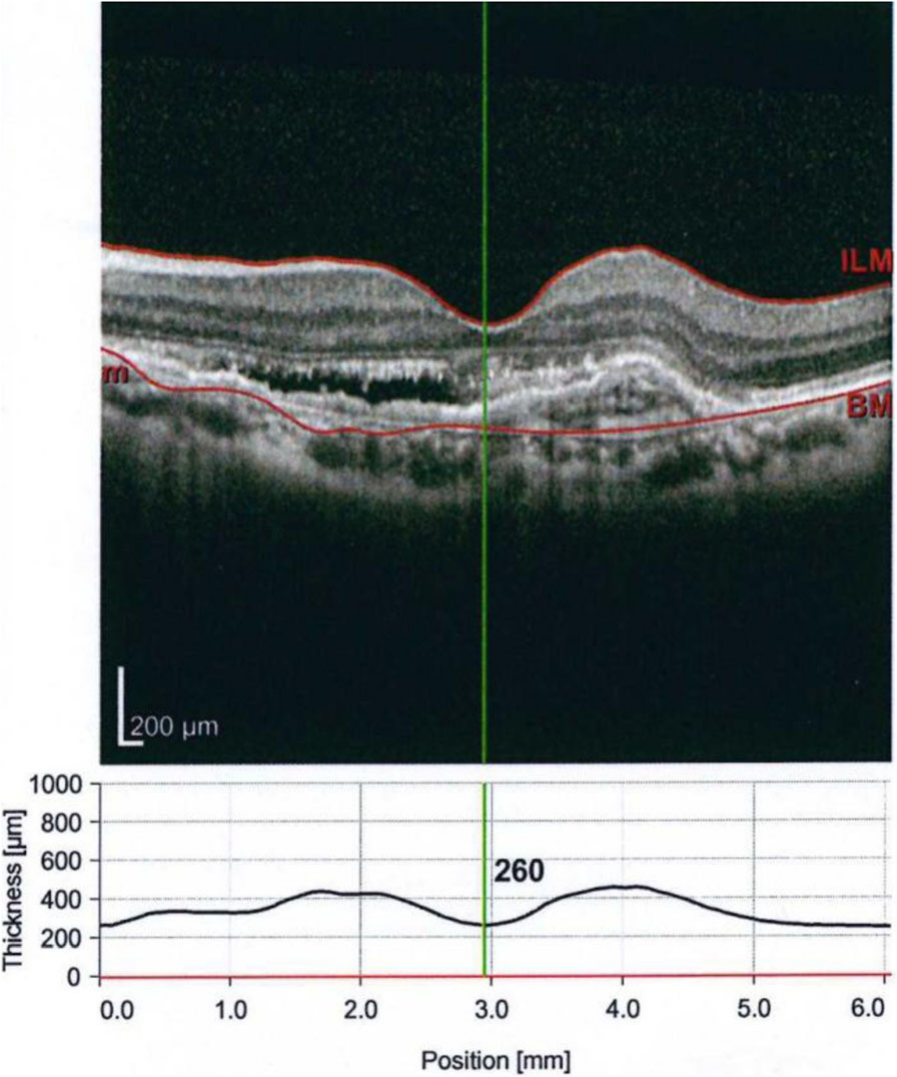
OCT of the same patient showing active subfoveal CNV, the CMT is 260 um

**Fig. 5:**
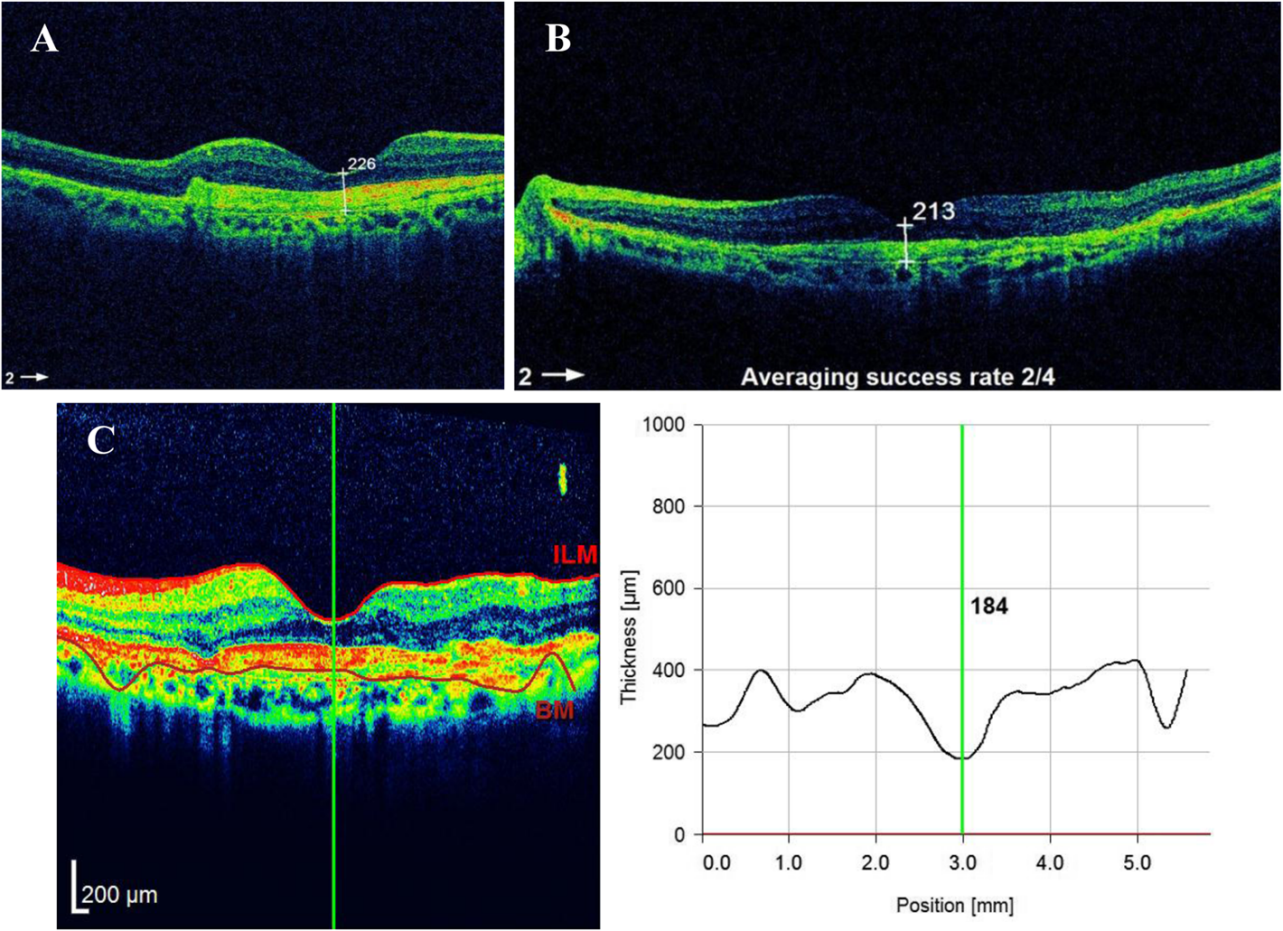
**a** OCT after the first injection of ziv-aflibercept with reduced CMT to 226 um. **b** OCT after the second injection of ziv-aflibercept with reduced CMT to 213um. **c** OCT after the third injection of ziv-aflibercept, the CMT declined to 184 um

Subconjunctival haemorrhage occurred in three eyes during injection. No cases of reaction, endophthalmitis, retinal breaks, retinal detachment, or vitreous haemorrhage were reported. No systemic complications, such as myocardial infarction, stroke or death, were reported during the study. For example, in a 55-year-old female patient with active myopic CNV and retinal haemorrhages as documented by fluorescein angiography (Fig. 3), BCVA improved after 3 consecutive injections, from 1 to 0.52 (by LogMAR), and CMT declined, from 260 μm (Fig. 4) to 226 μm, 213 μm, and 184 μm after 3 monthly injections (Fig. 5a, b, c). CNV remained inactive until the last follow-up visit.

## Discussion

Various methods, such as laser photocoagulation, transpupillary thermotherapy (TTT), PDT (photodynamic therapy), submacular surgery, and macular translocation, were attempted to treat myopic CNV before the era of anti-VEGF. Despite all these attempted treatment approaches, the results were unsatisfactory, with no evident clinical improvement. Damage to adjacent photoreceptors and atrophic scar progression hindered clinical improvements and decreased BCVA in laser photocoagulation-treated patients with subfoveal CNV due to age-related macular degeneration [12, 13], with even poorer results in patients with myopic extrafoveal CNV despite the cease of activity [14–17].

This advocated the shift to PDT with verteporfin, which resulted in more satisfactory results angiographically and clinically in myopic CNV patients. Many studies have supported the use of verteporfin, especially those reported by the Photodynamic Therapy Study Group, which detected an improvement in BCVA in the PDT-treated group in the first 2 years that declined afterward [4, 18]. Another study that included 24 eyes of myopic CNV patients reported that PDT may result in good visual outcomes in patients with extrafoveal CNV lesions when the laser spot is adjusted to spare the fovea [19].

Regarding anti-VEGF treatment for myopic CNV, Baba et al. [20] compared the efficacy of bevacizumab and PDT. Intravitreal bevacizumab showed a greater improvement in BCVA than PDT in eyes with myopic CNV. Additionally, Arias et al. [21] found that the mean visual acuity improved by 8.4 letters following an average of 1.5 intravitreal bevacizumab injections after 6 months of follow-up. Another study conducted by Ikuno et al. [22] reported an improvement in the mean BCVA in a case series of 63 eyes of myopic CNV following one to six bevacizumab injections.

during 12 months of observation. Regarding ranibizumab, the REPAIR study [23, 24] reported an improvement of 13.8 letters following a median of 3 ranibizumab injections based on the PRN regimen after 12 months. The RADIANCE study confirmed the results of the REPAIR study [8], which showed that approximately 40% of ranibizumab-treated patients gained 15 or more letters of visual acuity at the third month compared with only 15% of PDT-treated patients. After 1 year, BCVA improved by 13.8 letters in the first group treated with ranibizumab (based on visual acuity stabilisation in the preceding two follow-up visits) and 14.4 letters in the second group treated with ranibizumab (based on disease activity criteria) compared with 9.3 letters in the group treated with PDT. In addition, some patients in the PDT group switched to ranibizumab from the third month onwards (with a median number of 2.0 injections between months 3 and 12).

Another case series conducted on ranibizumab in 16 patients with myopic CNV by Lai et al. [25] reported an improvement in the mean BCVA of 3 lines after 1 year in 75% of eyes, with recurrence demonstrated by angiography in only two eyes after 3 months that required retreatment between the 3rd and 9th months. A significant reduction in the mean central foveal thickness by OCT was also reported.

Another study by Chen et al. [26] compared the efficacy of ranibizumab and PDT in myopic CNV patients and revealed that in Asian patients, ranibizumab achieved superior efficacy compared to verteporfin photodynamic therapy at the third month, with continuation of its therapeutic effect at month 12. Furthermore, a recent 5-year, prospective, multicenter study that was conducted on ranibizumab in myopic CNV in 488 clinical sites in 42 countries revealed tremendous VA gains in treatment-naive patients and VA maintenance in patients previously treated with ranibizumab [27].

With respect to aflibercept in myopic CNV, the MYRROR study reported a mean change of + 13.5 letters in BCVA in aflibercept-treated patients after a median of two injections within 12 weeks that was maintained throughout 48 weeks compared with + 3.9 letters in sham-treated patients. No further injections were needed throughout the study. Visual gain correlated significantly with a reduction in central retinal thickness [9]. Other studies confirmed the efficacy of aflibercept in myopic CNV patients. For example, Brue and co-authors showed an improvement in BCVA from 0.69 LogMAR to 0.15 LogMAR after 18 months [28]. Moreover, Pece and Milani reported an improvement of 10.6 letters after 1 year of follow-up [29].

The present study evaluated the efficacy and safety of ziv-aflibercept in myopic CNV patients and reported that fewer injections were needed in patients younger than 40 years. In addition, CNV was smaller in the younger age group, with a more notable improvement in BCVA. These results are similar to those of Yoshida et al. [30], who also showed more notable clinical improvements with a smaller CNV in patients younger than 40 years. To explain the less notable improvement in older patients, several factors, such as decreased integrity and function of the retinal pigment epithelium in myopic patients, may be involved, as these might reduce the inhibition of angiogenesis, leading to the formation of a larger and more active CNV, as well as a delay in the regression of CNV in older patients. Moreover, myopic CNV in older patients shows a clinicopathological correlation with both AMD and high myopia and tends to correlate with chorioretinal atrophic changes that markedly affect visual acuity [20]. Another study evaluated the influence of ethnicity and age on the response to ranibizumab injection in myopic CNV and revealed that East Asians showed better BCVA gains than Caucasians. Variable numbers of injections were given to the different subgroups, indicating the need for individualized treatment [31].

Additionally, the improvement in BCVA and the reduction in CMT after injection were achieved in both groups, with statistical significance that reflects the efficacy of the new drug in myopic CNV patients with a high safety profile and low cost. This finding was quite similar to those from recent studies performed on ziv-aflibercept on a larger number of patients [32, 33] that discussed the application of ziv-aflibercept in different retinal diseases and detected a reasonable safety profile for the drug comparable with those of other anti-VEGF agents. Moreover, Braimah et al. [11] studied the efficacy of ziv-aflibercept on retinal diseases other than AMD, such as high myopia, macular telangiectasia, central serous chorioretinopathy, choroidal osteoma, choroiditis, Best’s disease and idiopathic macular degeneration, and reported a marked reduction in CMT and a moderate improvement in BCVA.

In contrast, a study performed by Chhablani et al. on patients with age-related macular degeneration suggested immediate IOP elevation after injection in four eyes that needed anterior chamber paracentesis [34]. Anterior chamber paracentesis was not performed in the present study, as IOP did not change significantly after the injection of ziv-aflibercept either immediately or an extended period of time. This demonstrates the safety of ziv-aflibercept on IOP.

The costs per dose of intravitreal bevacizumab and ziv-aflibercept are low [35, 36]; however, those of aflibercept and ranibizumab are 20 to 30 times higher [37]. Thus, this drug can be of great benefit in low-income countries with deficient insurance coverage.

The main limitations of this study are the small number of patients, the short duration of follow-up (6 months) and the lack of a control group; thus, a larger number of patients and a longer follow-up period are needed to confirm our results.

## Conclusion

Ziv aflibercept is a cheap and effective anti-VEGF agent in patients with active myopic CNV, with a high safety profile. The authors recommend the use of this new anti-VEGF agent in retinal diseases such as myopic CNV. However, a larger number of patients and a longer follow-up period are needed in further studies to confirm these results.

## Data Availability

The datasets used during the current study are available from the corresponding author on a reasonable request.

## Abbreviations

AMD: Age-related macular degeneration
BCVA: Best corrected visual acuity
CMT: Central macular thickness
CNV: Choroidal neovascularisation
FFA: Fundus fluorescein angiography
IOP: Intraocular pressure
OCT: Optical coherence tomography
PDT: Photodynamic therapy
TTT: Transpupillary thermotherapy
VEGF: Vascular endothelial growth factor
